# Molecular Epidemiological Characteristics and Risk Factors for Acquiring HBV Among Li Ethnic in Baisha County, Hainan Island-Subgenotype D3 Was First Discovered in China

**DOI:** 10.3389/fmicb.2022.837746

**Published:** 2022-02-07

**Authors:** Ru Xu, Dandan Song, Min Wang, Jieting Huang, Qiao Liao, Zhengang Shan, Xia Rong, Yongshui Fu

**Affiliations:** ^1^Institute of Clinical Blood Transfusion, Guangzhou Blood Center, Guangzhou, China; ^2^The Key Medical Laboratory of Guangzhou, Guangzhou, China; ^3^Department of Clinical Laboratory, The Fifth People’s Hospital of Zhuhai, Zhuhai, China; ^4^School of Laboratory Medicine and Biotechnology, Southern Medical University, Guangzhou, China; ^5^Zhujiang Hospital of Southern Medical University, Guangzhou, China

**Keywords:** hepatitis B virus, risk factors, epidemiology, Li ethnic, HBV genotype

## Abstract

The residents of Baisha, a county of Hainan Island, mainly composed of Li ethnic population and relatively closed living environment with its unique geographical location. Our previous study showed that Li ethnic population of Baisha is an endemic center for hepatitis *C* virus, with significantly higher rates than in other parts of China. However, the epidemiology of HBV in this region remains unclear. Therefore, we conducted a comprehensive epidemiological survey of HBV in Baisha County, including 1,682 Li ethnic residents. The total seropositive rate for HBsAg was 10.2% and was higher than other parts of China. HBV-positive status was associated with the 20–40-year-old group (OR = 1.27, 95%CI 1.04–1.39, *P* < 0.01) and alcohol consumption (OR = 2.17, 95%CI 1.58–2.99, *P* < 0.01). Phylogenetic analysis showed that HBV subgenotype *D3* was predominant in Baisha County which was first discovered in China, followed by *C5*, *C1*, *B2*, and undetermined subgenotypes which were significantly different from other geographical distribution of main genotypes in China. The most recent common ancestor (tMRCA) of the *HBV genotype C* in the Li ethnic of Baisha County was 1846 (95%CI: 1739–1932), and *Baisha-C5* was earlier than *Baisha-C1* and *Baisha-C2*. Most *Baisha-D3* sequences were concentrated in one bundle and unrelated to those *D3* genome sequences elsewhere in the world. According to the phylogenetic tree, *D3* was introduced into Baisha County in 1884 (95%CI: 1816–1993) and became a local endemic virus. In conclusion, HBV infection in the Li ethnic group is characterized by a high prevalence rate in 20–40-year-old individuals and a unique genotype distribution which were significantly different from other geographical distribution of main genotypes in China, and subgenotype D3 was first discovered in China.

## Introduction

Hepatitis B virus (HBV) infection is a significant public health challenge, especially in developing countries with high prevalence. The epidemiology of HBV could be tracked by the prevalence of hepatitis B surface antigen (HBsAg), which declined from 9.8 to 7.2% in the general population aged 1–59 years in China (Xia et al., 1996; Liang et al., 2013). A modeling study showed that the prevalence of HBsAg was 6.1% in 2016 in mainland China, corresponding to 86 million infections (Polaris Observatory, 2018). Nevertheless, the prevalence of HBsAg was relatively low in the blood donors. For example, 1.16% was found from 1999 to 2009 in Xi’an blood donors (Zhao et al., 2014), 2.3% was found from 2005 to 2014 in Shenzhen blood donors (Wang et al., 2016); while in Guangdong, only 0.06% was found in repeat donors who were born after 1992 following nationwide implementation of universal HBV vaccination at birth (Tang et al., 2018). Baisha County is a mountainous rural area of Hainan Province that is located between Southeast Asia and East Asia close to Vietnam. Most native residents belong to the Li ethnic population (Li et al., 2008). We have reported the unique genetic background (HLA class I and II alleles) of the Baisha Li ethnic, which was distinct from Chinese Han ethnicity (Huang et al., 2019). Our recent study showed that Baisha County is an endemic center for HCV infection, where the rate of anti-HCV was significantly higher than other areas in China (Xu et al., 2019). However, the prevalence of HBV infection remains unclear in this county. Some reports have shown that the prevalence of HBV was higher in rural areas than in urban areas (Zhang et al., 2013; Liu et al., 2017; Wang et al., 2019). Two studies also reported that the seroprevalence of HBV infection was higher in Hainan Province than in other parts of China (Li et al., 2008; Sun et al., 2008). Therefore, conducting an epidemiological investigation of HBV in this special region is not only vital to understand the burden of HBV infection and to predict the future prevalence of HBV in the Hainan Li ethnic population, but also helpful to understand the cause of the distinctive HBV epidemiology in this area.

Hepatitis B virus is divided into 10 genotypes (A-J), and the geographical distribution of each genotype was distinct, even in the same country (Sunbul, 2014). The HBV genotype *B* was primarily distributed in the south and genotype *C* in the north of China (Lin et al., 2013; Li et al., 2017). Genotype *D* was endemic to northwestern China (Nie et al., 2012). *C/D* recombinant genotype was found in Qinghai-Tibet Plateau in Western China and predominantly in Tibet (Zhou et al., 2011). Genotypes *E, F, G, H* and *J* have not been reported in China (Su et al., 2020). A study showed that HBV vaccination did not interfere with HBV genotype distribution (Su et al., 2020). However, different genotypes are related to clinical progression, response to antiviral treatment, and prognosis (Yuen et al., 2018). HBV genotype can influence the clinical outcome of HBV infection. Mayerat et al. (1999) found that there is a clear relationship between genotype *A* and chronic infection when compared to genotype *D*. By contrast, studies have revealed that the clinical outcomes of chronic HBV infections are more serious in patients with genotypes *C* and *D* than in those with genotypes A and B. Furthermore, the patients with genotypes *C* and *D* were prone to cirrhosis and hepatocellular carcinoma (HCC), the risk of cirrhosis and HCC in patients with genotype *F* infection was similar to genotypes *C* and *D* (Shi, 2012). HBV genotypes can also influence the response to antiviral treatment of HBV infection. For example, genotypes *A* and *B* have higher response to interferon-based therapy than genotypes *C, D* and mixed genotypes. A study showed that genotype *A* was a 20-fold increase in the risk of nucleos(t)ide analogs resistance when compared to that of genotype *D* for a mean period of 12 months (Maria et al., 2002). In addition, the characterization of different HBV genotypes in a given population may have epidemiological importance, as the HBV genotype generally reflects its country of origin and can be used to track transmission patterns.

In this study, we focused on the molecular epidemiological characteristics and risk factors for HBV infection in Baisha County in southernmost China. We found that the HBV infection rate was high, and there were unique genotypes, especially D3, which was the first subgenotype discovered in China. We speculated that the closed geographical location and the unique living habits of Li ethnic played an important role in the local transmission of the virus. Such information could help improve HBV prevention and control strategies in this county and potentially avoid transmission to other regions of Hainan Island. Moreover, it could give insight into the evolution and origin of HBV in the Li ethnic population.

## Materials and Methods

### Sample Collection

From July 2014 to October 2015, 1,682 11–95-year-old volunteers from four communities and seven townships in Baisha County were recruited in this study by a random sampling method, the same cohort as our previous study (Xu et al., 2019). The participants’ socio-demographic features and history of blood transfusion, alcohol consumption, surgery, acupuncture, tattoos, drug abuse, body piercings, dental operations, and HBV infection among family members were recorded. Then, blood samples were collected to assess HBV markers. The physicians ensured that individuals were personally interviewed to assure their complete understanding of the study, and the participants provided written informed consent before enrollment. The Institutional Review Board approved this study at the Guangzhou Blood Center, and the guidelines set by this board were strictly followed. All the study protocols conformed to the 1975 Declaration of Helsinki’s ethical guidelines and were approved by the Medical Ethics Committee of the Guangzhou Blood Center.

### Hepatitis B Surface Antigen and Hepatitis B Virus DNA Detection

According to the manufacturers’ instructions, the 1,682 plasma specimens were tested for HBsAg using two independent HBV ELISA assays (Beijing Wantai, China and Monolisa HBsAg ULTRA, Bio-rad, United States). The HBV DNA levels were assessed using an in-house combination of qPCR, with the lowest detection limit being 5 IU/ml, as described previously (Zheng et al., 2015).

### PCR Amplification and Sequencing

According to the manufacturers’ instructions, HBV DNA was extracted from 250 μL plasma samples using the MagNA Pure LC Nucleic Acid Isolation Kit-Large Volume (Roche Diagnostics). A nested PCR performed amplification with a partial P region primer from the protocol available at the GOV.UK website^1^, (primers were shown in Supplementary Table 1). The final amplicon was approximately 1 kb in length and covered the entire HBsAg region (Position: nucleotides 1–1,095, numbered according to with HBV genotype *B* and *C*). If the partial P region was unsuccessfully amplified, part of Pre-S/S (Position: nucleotides 1–423 and 2,817–3,215, numbered as before) were independently amplified according to a previous study (Nie et al., 2012) (primers were shown in Supplementary Table 1). Eleven highly viremic serum samples of the subgenotype *D3* were performed to obtain whole genomes by other two fragments (Position: nucleotides 3,194–1,797 and nucleotides 1,777–274) primers which were shown in Supplementary Table 2. The amplified products were purified and sequenced by Beijing Genomics Institute (BGI, Beijing, China). The obtained sequences were then inspected and aligned using the SeqMan program from DNASTAR^2^ and BioEdit software^3^.

### Genotype and Subgenotype Analysis

Hepatitis B virus genotypes and subgenotypes were classified directly from GenBank sequence annotations and phylogenetic trees. The phylogenetic tree was constructed based on the maximum-likelihood method using MEGA-X software^4^. The reliability of the tree was estimated using 1,000 bootstrap replications. Bootstrap values of the phylogenetic branches greater than 70% were considered as having a high degree of confidence. Simplot programs (available at^5^) were used to test for recombination. The reference sequences covering HBV genotypes A-J and major subgenotypes were obtained from GenBank and are published in peer-reviewed journals.

### Hepatitis B Virus Genotype/Subgenotype Dataset

*Hepatitis B Virus-C* and *HBV-D* partial P region sequences were retrieved from the continent available in GenBank to identify the *HBV-C* and *HBV-D* subgenotypes circulating in Baisha County and determine their transmission and origin. Sequences without a known County, sampling date, and less than 1 kb were excluded. To avoid the over-representation of unrelated Baisha sequences, we built a phylogenetic tree using MEGA-X to exclude unrelated Baisha reference sequences and very close reference sequences.

### Evolutionary Rates and Divergence Dates

To co-estimate evolutionary rates, timescale phylogeny, and model parameters, we used the Bayesian Markov chain Monte Carlo (MCMC) method implemented in the BEAST v1.10.4 (Bayesian Evolutionary Analysis by Sampling Trees) package with strict, lognormal, and exponential clock model under a less restrictive Bayesian skyline plot (BSP) coalescent model. Eventually, the GTR + G + I nucleotide substitution and lognormal clock models were chosen as the best models for analysis. The MCMC chains were run for at least 200 million generations and sampled every 2,000 generations. Convergence was assessed based on the effective sampling size (ESS) after a 10% burn-in using Tracer software version 1.5; only ESS values above 200 were accepted. The reconstructed trees were examined and edited using FigTree v1.4.0, which was also used to estimate various nodes’ evolutionary rates and dates on the MCMC tree.

### Statistical Analysis

A univariate analysis used the chi-square test to detect associations between HBV infections and participants’ socio-demographic characteristics/risk factors. Measurement data were presented as the mean ± standard error and compared using the two-sample *t*-test. A multivariate logistic regression analysis was performed to determine the predictors of HBsAg positivity. Furthermore, data were expressed as the mean (standard deviation; SD), percentage (%), and OR (95% confidence interval; CI), where appropriate; *P* < 0.05 was considered statistically significant. All statistical analyses were performed using SPSS Statistics for Windows version 19.0 (IBM Corp., Armonk, New York).

### Nucleotide Sequence Accession Numbers

The nucleotide sequences reported in this study were deposited in GenBank with the following accession numbers MW244442-MW244564 and MW575198-MW 575215.

## Results

### Hepatitis B Virus Infection in the Baisha Li Ethnic Population

Total of 1,682 blood samples from the Baisha Li group were tested for HBsAg, 171 (10.2%) and 1,465 (87.0%) were reactive and non-reactive, respectively, with two independent assays (Table 1). In addition, 30 (1.8%) were only reactive with Monolisa HBsAg ULTRA from Bio-Rad, 16 (1.0%) were only reactive with the HBV ELISA assay from Wantai. 149 HBV DNA + was detected in 171 HBsAg + samples with a mean viral load of 4.67 × 10^4^ ± 223 IU/ml.

**TABLE 1:** Seroprevalence of hepatitis B virus infections of Li ethnic.

Monolisa HBsAg ULTRA from Bio-Rad	HBV ELISA Assay from Wantai		Total
	Reactive	Non-reactive	
Reactive	171 (10.2)	30 (1.8)	201 (12.0)
Non-reactive	16 (1.0)	1,465 (87.0)	1,481 (88.0)
Total	187 (11.1)	1,495 (88.9)	1,682 (100.0)

### Risk Factors for Hepatitis B Virus Infection

Hepatitis B surface antigen positivity was significantly higher in 20–40-year-old (24.6%) and 40–60-year-old (14.1%) subjects compared to younger (10–20 years old, 5.8%) or older subjects (> 60 years old, 3.8%) in our series (χ^2^ = 1.07E2, d.f. = 4, *P* < 0.01, chi-square test). The detailed results of the comparison between the two groups (20–40 years old and any other age groups) are shown in Table 2. A logistic regression analysis, with adjusted confounders, confirmed that age was a significant predictor for HBV positivity (OR = 1.27, 95%CI 1.04–1.39, *P* < 0.01). There was a significant increase in the HBsAg positivity in male than female subjects (OR = 1.81, 95%CI 1.32–2.50, *P* < 0.01, chi-square test) (Table 2). Alcohol consumption was more common in HBsAg positive group than in HBsAg negative group (OR = 2.17, 95%CI 1.58–2.99, *P* < 0.01; chi-square test), while there was no difference in transfusion, drug abuse, piercings, acupuncture, tattooing, surgery, dental procedures, or a family history of HBV between HBsAg positive group and HBsAg negative group. A logistic regression analysis, with adjusted confounders, confirmed that alcohol consumption (OR = 2.97, 95%CI 2.03–4.33, *P* < 0.01) was a significant predictor for HBV positivity (Table 3).

**TABLE 2:** Socio-demographics of subjects according to HBsAg positivity.

	HBsAg (+) *n* = 171	HBsAg (-) *n* = 1,511	Univariate Chi-square analysis		Multivariate logistic regression analysis	
			OR (95%CI)	*P*-value	OR (95%CI)	*P*-value
**Gender**						
Male	**89 (13.6)**	565 (86.4)	**1.81 (1.32**–**2.50)**	**1.95E-04**	1.06 (0.72–1.56)	0.77
Female	**82 (7.98)**	946 (92.02)				
**Age**						
< 20y	17 (5.8)	278 (94.2)	**0.19 (0.06–0.19)**	**2.42E-10**	**1.27 (1.04–1.39)**	**1.52E-07**
20–40y	68 (24.6)	208 (75.4)	**1**	**-**		
40–60y	60 (14.1)	366 (85.9)	**0.50 (0.34–0.74)**	**4.05E-4**		
> 60y	26 (3.8)	659 (96.2)	**0.12 (0.08–0.19)**	**7.51E-23**		

*P-values less than 0.05 are indicated in bold.*

**TABLE 3:** Risk factors for HBV infection.

Risk Factors	HBsAg (+) *n* = 171	HBsAg (–) *n* = 1,511	Univariate Chi-square analysis		Multivariate logistic regression analysis	
			OR (95%CI)	*P*-value	OR (95%CI)	*P*-value
**Alcohol consumption**						
Yes	98 (14.5)	577 (85.5)	**2.17 (1.58**–**2.99)**	**1.33E-06**	**2.97 (2.03**–**4.33)**	**1.73E-08**
No	73 (7.2)	934 (92.8)				
**Drug abuse**						
Yes	0 (0)	3 (100)	0.99 (0.99–1.00)	0.99*	-	0.99
No	171 (10.2)	1,508 (89.8)				
**Tattooing**						
Yes	7 (5.2)	128 (94.8)	0.46 (0.21–1.004)	0.052	0.84 (0.36–1.93)	0.68
No	164 (10.6)	1,383 (89.4)				
**Surgery**						
Yes	3 (5.5)	52 (94.5)	0.50 (0.16–1.62)	0.24	0.38 (0.09–1.52)	0.17
No	168 (10.3)	1,459 (89.7)				
**Piercings**						
Yes	38 (8.9)	388 (91.1)	0.83 (0.57–1.21)	0.33	1.11 (0.71–1.74)	0.64
No	133 (10.6)	1,123 (89.4)				
**Acupuncture**						
Yes	1 (3.6)	27 (96.4)	0.32 (0.04–2.39)	0.24	0.48 (0.05–4.22)	0.50
No	170 (10.3)	1,484 (89.7)				
**Dental procedures**						
Yes	7 (16.3)	36 (83.7)	1.75 (0.77–3.99)	0.18	2.47 (0.99–6.06)	0.051
No	164 (10.0)	1,475 (90.0)				
**Guasha**						
Yes	0 (0)	8 (100)	0.99 (0.991–0.998)	0.99*	-	0.999
No	171 (52.8)	1,503 (47.2)				
**Transfusion**						
Yes	5 (17.2)	24 (82.8)	1.87 (0.70–4.96)	0.20	2.08 (0.65–6.64)	0.22
No	166 (10.0)	1,487 (90.0)				
**A family history of HBV infection**						
Yes	2 (22.2)	7 (77.8)	2.54 (0.52–2.34)	0.23	4.26 (0.73–24.86)	0.11
No	169 (10.1)	1,504 (89.9)				

*Data were obtained by Fisher’s exact test. P-values less than 0.05 are indicated in bold.

### Genotype/Subgenotype Distribution of Baisha Hepatitis B Virus Strains

**FIGURE 1:**
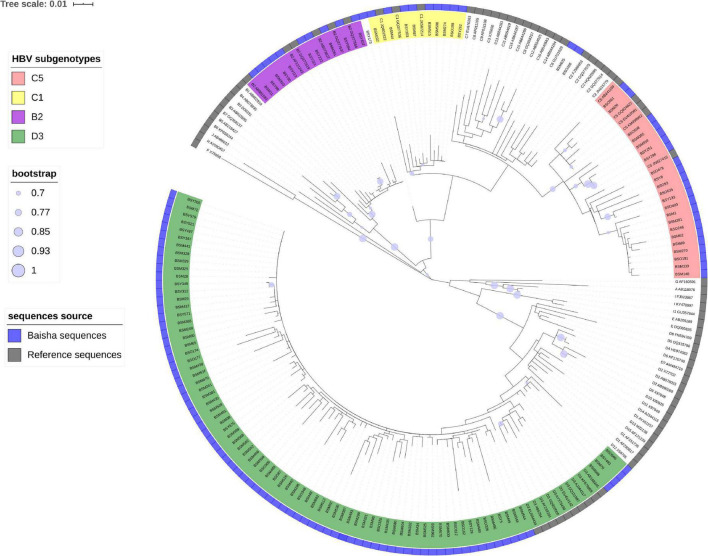
Estimated maximum-likelihood phylogeny for Baisha’s Li ethnic partial P region sequences. The sequences identified in this study are shown in a blue strip. Reference sequences were named by subgenotype, and accession numbers represent the assigned HBV genotypes and subgenotypes.

**FIGURE 2:**
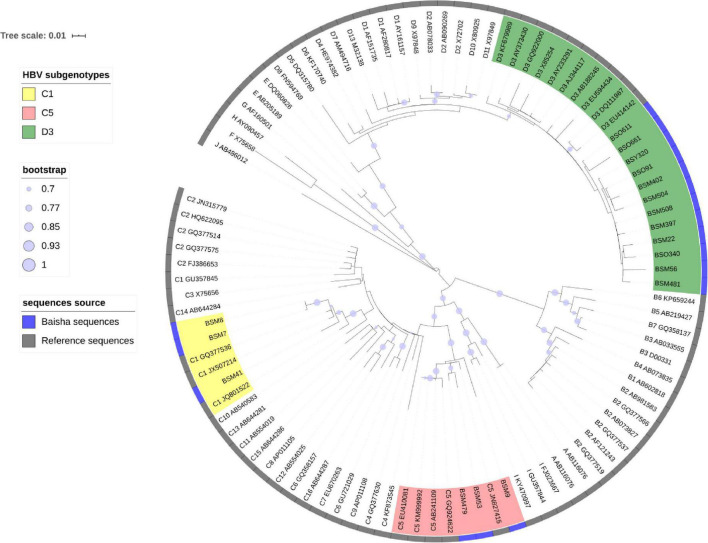
Estimated maximum-likelihood phylogeny for Baisha’s Li ethnic part of Pre-S/S region(1b) sequences. The sequences determined in this study are shown in a blue strip. Reference sequences were named by subgenotype, and accession numbers represent the assigned HBV genotypes and subgenotypes.

Nested PCR amplification was performed on HBV DNA + specimens with different PCR primers. A total of 141 DNA fragments were successfully amplified in 149 HBV DNA + cases, including partial P region (nt 1–1,095) of 123 cases and PreS/S (nt 2,817–3,215 and 1–423) of 18 cases. Neither of the two fragments were amplified in eight samples because of the very low viral loads or limited plasma volumes. The phylogenetic tree constructed from 123 partial P region sequences showed that 79 cases were *D3*, 10 were *B2*, 22 were *C5*, 9 were *C1*, 2 were new subgenotypes of genotype *C*, and 1 (*BSY173*) was a possible recombinant of genotype *C* and *X* (Figure 1). The phylogenetic tree of 18 partial Pre-S/S genes showed that 12 cases were *D3*, 3 were *C5* and 3 were *C1* (Figure 2). Eleven HBV subgenotype D3 full-length sequences were genotyped to exclude the possibility of *C/D* hybrid genotypes that reported from the Tibetan ethnic populations living in Northwest China (Cui et al., 2002; Wang et al., 2005). The tree showed that a pure genotype *D* circulated among the Li ethnic populations (Supplementary Figure 1). Furthermore, Simplot also did not show any recombination events (data not shown).

### Evolutionary Analyses of Genotypes *C* and *D*

Genotypes *C* (*HBV-C*) and *D* (*HBV-D*) were the main HBV genotypes detected on the Li ethnic in Baisha County (Figure 1). Thus, we performed an evolutionary analysis of *HBV-C* and *HBV-D* separately in Baisha County with the global reference sequences. *BSY173* was excluded because the possible recombinant events would strongly affect the estimates of both the nucleotide substitution rate and the age of genetic diversity (Zhou and Holmes, 2007).

**FIGURE 3:**
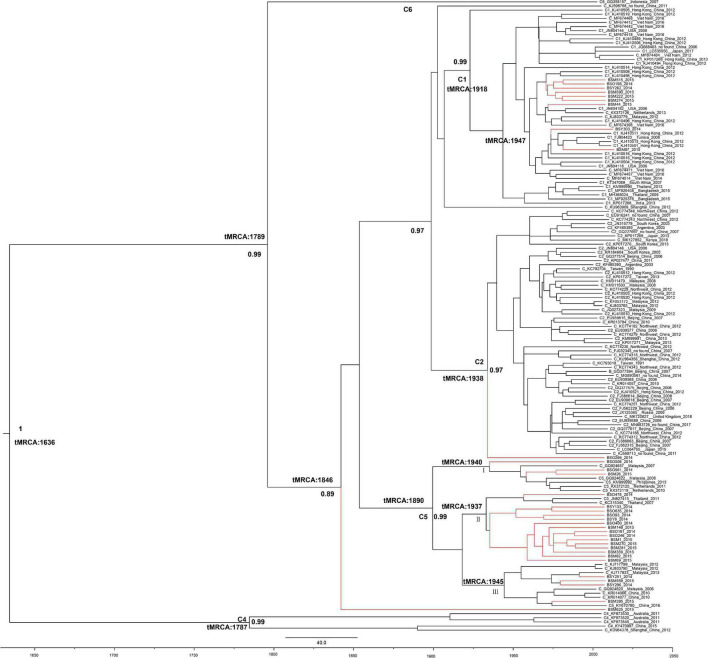
Bayesian maximum clade tree of *HBV-C* partial P region sequences. Branches of Baisha sequences are colored red. The scale at the bottom of the tree represents years before the last sampling time (2015). All nodes marked with an asterisk show a posterior probability > 0.90. The tree was automatically rooted under the assumption of a relaxed molecular clock.

The evolutionary rate of *HBV-C* was 2.29 × 10^–4^ substitutions per site per year (s/s/y, 95%CI: 5.99 × 10^–5^, 4.10 × 10^–4^). The most recent common ancestor (tMRCA) of the *HBV-C* in the Li ethnic of Baisha County was from the year 1846 (95%CI: 1739–1932). *BSM625* (an undetermined subgenotype) was the most ancient sequence in Baisha County, since it located in the root of all Baisha sequences (Figure 3). The other sequences formed subgenotypes *C1*, *C5*, and an undetermined subgenotype (*BSO266*). There were three transmission routes when *C5* was introduced in 1890 (95%CI: 1739–1972) in Baisha County. Group I clustered with the references collected from Malaysia, the Philippines, and Netherlands. Most of the *C5* sequences clustered with Thai reference sequences (Group II), while others were related to China and Malaysia (Group III). *C1* may have originated from Hong Kong or Vietnam in 1918 (95%CI: 1765–1965) according to the tree topology. *BSO266* was located in the root of C2, which was traced to 1938 (95%CI: 1805–1997).

**FIGURE 4:**
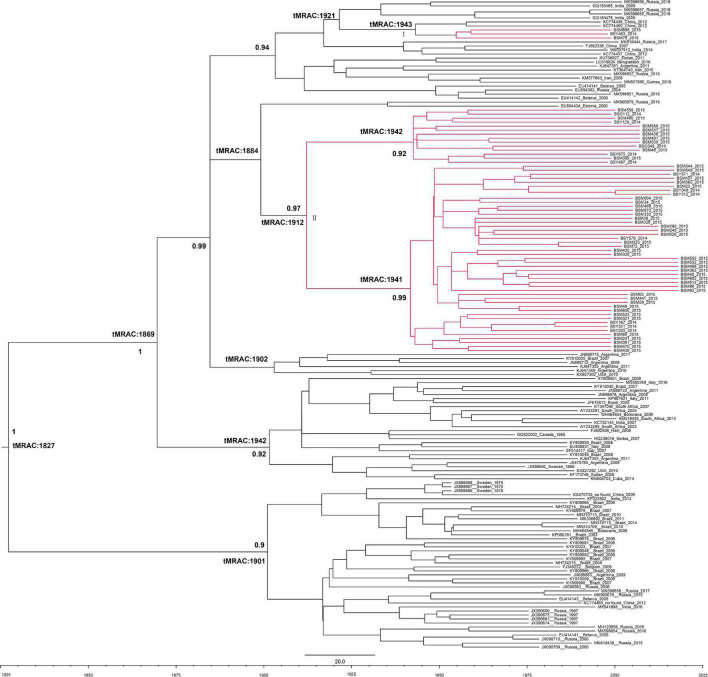
Bayesian maximum clade tree of *HBV-D3* partial P region sequences. Branches of Baisha sequences are colored red. The scale at the bottom of the tree represents years before the last sampling time (2015). All nodes marked with an asterisk show posterior probability > 0.90. The tree was automatically rooted under the assumption of a relaxed molecular clock.

The evolutionary rate of *HBV-D3* was 9.69 × 10^–5^s/s/y (95%CI: 3.39 × 10^–5^, 1.62 × 10^–4^). The tMRCA of subgenotype *D3* worldwide was from around 1827 (95%CI: 1673–1975), whereas most *D3* introduced in Baisha County was from around 1884 (95%CI: 1816–1993). The Baisha sequences formed two groups (I and II), indicating two distinct routes of introduction in Baisha County. Group II contained most of the sequences and was further divided into two branches, suggesting the two transmission events with tMRCA of the main clusters calculated as 1942 (95% CI: 1768–1963) and 1941 (95% CI: 1762–1968), respectively. In addition, as shown in the tree’s topology (Figure 4), Group II was unique, Baisha-specific, and may have originated from Russia and Estonia around 1912 (95%CI: 1847–2010). The Baisha sequences clustered in Group I were very close to two references which collected from northeast China and originated in 1943 (95%CI: 1884–1991), and possibly from Russia and India in 1921 (95%CI: 1865–2001) according to the tree’s topology.

## Discussion

The prevalence of HBV infection differs across China (MacLachlan and Cowie, 2015). Baisha County, located in Hainan Province of China’s southernmost, is the main residential area of the Li ethnic population. This area is isolated by the surrounded mountains at the entrance to East Asia and have distinctive genetic characteristics (Li et al., 2008). In this study, the HBsAg prevalence was found as high as 10.2%, suggesting that Baisha County was a highly endemic region according to the three categories of endemicity: high (> 8%), intermediate (2–7%) and low (< 2%) (Li et al., 2008). Epidemiological studies from 2006 to 2016 showed that HBV prevalence decreased from 7.2 to 3.8% (Liang et al., 2013; Zhang et al., 2016; Wang et al., 2018). A cross-sectional study showed that the overall HBsAg prevalence of rural childbearing-aged women was 9.51% in Hainan Province (Zhang et al., 2013). Further study showed that the prevalence of HBsAg in the Li ethnic population (10.56%) was higher than in the Han ethnic populations (9.08%) (Zhang et al., 2013), which was consistent with our study and higher than the prevalence in other areas in China. In previous study, a high HCV prevalence (7.0–9.1%) was found in the same cohort (Xu et al., 2019). However, no HBV/HCV co-infection was found, which was inconsistent with the results that HBV/HCV coinfection is not uncommon in highly endemic areas because of the shared modes of transmission (Dimitris and Melanie, 2015). We speculated that the different transmission routes were contributed to mono-infection with either HBV or HCV. In this study, the risk factors for HBV infection were 20–40-year-old group and alcohol consumption, while the risk factors for HCV infection were older age (≥ 60 years) and surgery (Xu et al., 2019).

We also found that HBsAg positivity was higher in 20–40-year-old and 40–60-year-old subjects compared to younger and older subjects, consistent with the results previously reported in Turkey, Korean and Togo (Yun et al., 2008; Min et al., 2013; Tozun et al., 2015; Kolou et al., 2017). The higher HBV infection in the 20–60-year-old range derives from lifestyles and behaviors that increase exposure to infections, such as alcohol consumption and sexual relationship. Sexual transmission was shown to be involved in HBV transmission (Nelson et al., 2016). Another possibility to explain the low prevalence observed in over 60 years old subjects is that some of them may die from HBV related complications after progression of chronic infection for years. Chronic HBV infection is considered to significantly increase the risk of liver cancer and the mortality rate of which ranks first among all kinds of malignant tumor in Hainan province especially in Baisha County where Li ethnic has inhabited for centuries (Wu et al., 2015). In addition, liver cancer is the third most common causes of life expectancy loss in Hainan Province (Dou et al., 2021). Effective vaccination can explain the lower prevalence in 10–20-year-old compared to 20–60-year-old subjects. Alcohol consumption was a significant independent determinant of HBsAg positivity. The Li ethnic populations living in the Baisha County commonly consume alcohol, especially middle-aged subjects. Szabo et al. showed that alcohol use was associated with reduced host defense (Szabo and Mandrekar, 2009). Another study showed that HBV replication was enhanced by alcohol consumption with a sevenfold increase in HBsAg and viral DNA levels (Chan and Levitsky, 2016). However, little is known about the potential role of alcohol in HBV infection. Some reports have indicated an association between male gender and greater HBsAg positivity (Kong et al., 2014; Ikezaki et al., 2016). In our study, no correlation between gender and HBV infection in multivariate analysis was found, although the univariate chi-square test showed more male patients to be HBsAg-positive. We speculated that this might due to the strong correlation between gender and alcohol consumption (χ^2^ = 85.37, OR = 0.39, 95%CI 0.32–0.48, *P* = 2.48E-20, data not shown) and the rates of alcohol consumption in male was significantly higher than in female [male (51.3%) vs. female (48.7%): χ^2^ = 80.06, *P* < 0.01, data not shown].

The mainly prevalent HBV genotypes in China are *B* and *C* (Li et al., 2015). Genotype *C* was predominant in northern China, while genotype *B* was mainly distributed in southern China (Yin et al., 2010; Li et al., 2015; Shen et al., 2015). In addition, *C/D* recombinants have been identified in northwest China (Zhou et al., 2011; Wang et al., 2014). Interestingly, the genotype distribution of the Li ethnic in Baisha County differed dramatically from any other regions of China. Even though Baisha County is located in southern China, genotype *B* is the lowest (7.1%). The proportion of genotype *C* (28.4%) in the Li ethnic was higher than that of genotype *B*, which was consistent with previous reports in Hainan Island (Zeng et al., 2009; Wang et al., 2014). Meanwhile, the subgenotypes of genotype *C* in Baisha County were significantly different from those of other regions. In Baisha, *C5* was the main endemic subgenotype, followed by *C1*. *C5* was not found in China except for three cases from Yunnan Province (Wang et al., 2014). Notably, the genotype *D* was the dominant genotype in the Li ethnic of Baisha County, all of which were subgenotype *D3*. The genotype *D* was reported only in a few areas in China, such as Xinjiang, Hainan, Gansu, and Shenyang (Ma et al., 2011; Nie et al., 2012; Zhang et al., 2015; Pu et al., 2016). For example, China’s investigation of HBV genotypes in Xinjiang found that most of the Xinjiang Uygur population infected with genotype *D* (64.2%), and all were *D1* subgenotype (Nie et al., 2012). Although the genotype *D* was found in Shenyang (28%) (Ma et al., 2011), Hainan Island (11.94%) (Zeng et al., 2009) and Gansu (47.19%) (Zhang et al., 2015), none of these sequences have any detailed subgenotype information in text and have not been submitted to websites, so we do not know whether they were *D3*. We speculated that this unique HBV genotype distribution pattern relates to the location of Baisha County, to be specific, a mountainous rural area on Hainan Island that isolated from mainland China. The native residents in Baisha belong to the Li ethnic that derived from a subgroup of Austronesians. The unique characteristics of this cohort may have a unique HBV transmission pattern and origin.

Therefore, we performed genetic evolution analyses on the specific genotypes *C* and *D* to trace the HBV transmission and evolution in Baisha County. The genotype *C* phylogenetic tree showed that the Li ethnic’s HBV-*C* genome sequences in Baisha County was mainly distributed into two clusters (*C1* and *C5*) and were associated with other *C* genome sequence branches in the world. The tMRCA of the *HBV-*C of the Li ethnic was from about the year of 1846 (95% CI: 1739–1932), which was later than China’s Opium War in 1840. The population and trade exchanges between Hainan Island and Southeast Asia can be traced back to ancient times, especially after 1840, when China gradually relaxed its foreign policy restrictions under pressure from European and American countries, allowing migration exchange between Hainan Island and Southeast Asia to a large scale. A previous study showed that genotype *C5* might have originated from Southeast Asia (Zhu et al., 2015). However, there is no clear evidence to support this point in the current study as all Baisha *C5* sequences were dispersed in references from Southeast Asia such as Thailand, Malaysia and Philippines, suggesting that there have been exchanges occasionally between Baisha County and Southeast Asia. Nevertheless, according to the tree’s topological structure in this study, Baisha *C1* clustered with sequences from Hong Kong that may have originated from Southeast Asia. Here in our results, Baisha *C5* (1890) was earlier than *C1* (1918) and *C2* (1938), which was in agreement with the previously estimated divergence times for these subgenotypes (Paraskevis et al., 2013).

The subgenotype *D3* BEAST tree showed that only four sequences of the Li ethnic in Baisha County were isolated (Group I), while all other sequences were concentrated in one bundle and were not related to *D3* genome sequences elsewhere in the world (Group II). The analysis showed that most subgenotype *D3* entered the Li ethnic of Baisha County in about 1912 (95% CI: 1847–2010) and became endemic. This event occurred later than the time when subgenotype *D3* entered in Argentina and Brazil (Spitz et al., 2019) which was supported by the tree’s topology in this study, as Argentina and Brazil were closer to the tree’s root (Figure 4). Most Baisha *D3* sequences formed a Baisha-specific group, suggesting that *D3* was introduced from Russia and Estonia and formed a local endemic. There is no population geographic evidence to support such transmission events. We speculated that the lack of linkage reference sequences in GenBank failed us to explain how D3 in Baisha originated from Russia or Estonia. More sequences from different regions and countries are needed to perform evolutionary analysis. Another small branch of Baisha *D3* was close to the reference sequences collected from China, India, and Russia, indicating that they entered Baisha County by cross-contact between these countries, but did not become endemic for some reasons.

To our knowledge, it is the first study regarding the epidemiology of HBV in the Li ethnic population of Baisha County in Hainan Island, where was also an endemic center for HCV. In addition, the current study also discovered HBV subgenotype *D3* in China for the first time. Our results were important for understanding the molecular epidemiological characteristics and risk factors of HBV in the Li ethnic population in China. Whether these observations are generalizable to populations outside of Hainan Island should be considered in the further study. Therefore, more samples from other ethnic population and other regions outside Baisha County even outside of Hainan Island are required to further conduct a more comprehensive analysis and to better understand the epidemiology of HBV among different ethnic populations across China.

## Conclusion

The current study revealed that the seropositive rate for HBsAg was 10.2% among Li ethnic population in Baisha County, Hainan Island. HBV-positive status was associated with 20–40-year-old subjects and alcohol consumption. The most prevalent HBV subgenotype D3 was first discovered in China, followed by C5, C1, B2 and undetermined subgenotypes. The distribution of HBV genotype among Li ethnic in Baisha County was unique compared with other regions in China. The tMRCA of the HBV genotype C in the Li ethnic was 1846 (95%CI: 1739–1932), and *Baisha-C5* was earlier than Baisha-C1 and Baisha-C2. Most D3 sequences were concentrated in one bundle and unrelated to those D3 genome sequences elsewhere in the world, which was introduced into Baisha County in 1884 (95%CI: 1816–1993) and became a local endemic virus.

## Data Availability Statement

The datasets presented in this study can be found in online repositories. The names of the repository/repositories and accession number(s) can be found in the article/Supplementary Material.

## Ethics Statement

The studies involving human participants were reviewed and approved by the Medical Ethics Committee of Guangzhou Blood Center. Written informed consent to participate in this study was provided by the participants’ legal guardian/next of kin.

## Author Contributions

YF, XR, and RX conceived the study. RX, DS, MW, and JH conducted the study. RX, DS, QL, and ZS analyzed the data. RX and DS wrote the manuscript. JH, XR, YF, and RX revised and finalized the manuscript. All authors have read and agreed to the published version of the manuscript.

## Conflict of Interest

The authors declare that the research was conducted in the absence of any commercial or financial relationships that could be construed as a potential conflict of interest.

## Publisher’s Note

All claims expressed in this article are solely those of the authors and do not necessarily represent those of their affiliated organizations, or those of the publisher, the editors and the reviewers. Any product that may be evaluated in this article, or claim that may be made by its manufacturer, is not guaranteed or endorsed by the publisher.

## Footnotes

1
https://assets.publishing.service.gov.uk/government/uploads/system/uploads/attachment_data/file/342414/cfi_vrd_PolHBsAgRecommendationsFINAL_HBV_protocol.pdf

2
https://www.dnastar.com/

3
https://bioedit.software.informer.com

4
https://www.megasoftware.net/

5
https://simplot.software.informer.com/

## Supplementary Material

Supplementary Figure 1 Estimated maximum-likelihood phylogeny for Baisha’s Li ethnic HBV whole-genome sequences. The sequences identified in this study are labeled by “◆.” Others are reference sequences.
