# Supplementary figures and images for: Molecular Epidemiological Characteristics and Risk Factors for Acquiring HBV Among Li Ethnic in Baisha County, Hainan Island-Subgenotype D3 Was First Discovered in China
Source: Front Microbiol. 2022 Feb 7;13:837746. doi: 10.3389/fmicb.2022.837746 (PMC8859303; doi:10.3389/fmicb.2022.837746)

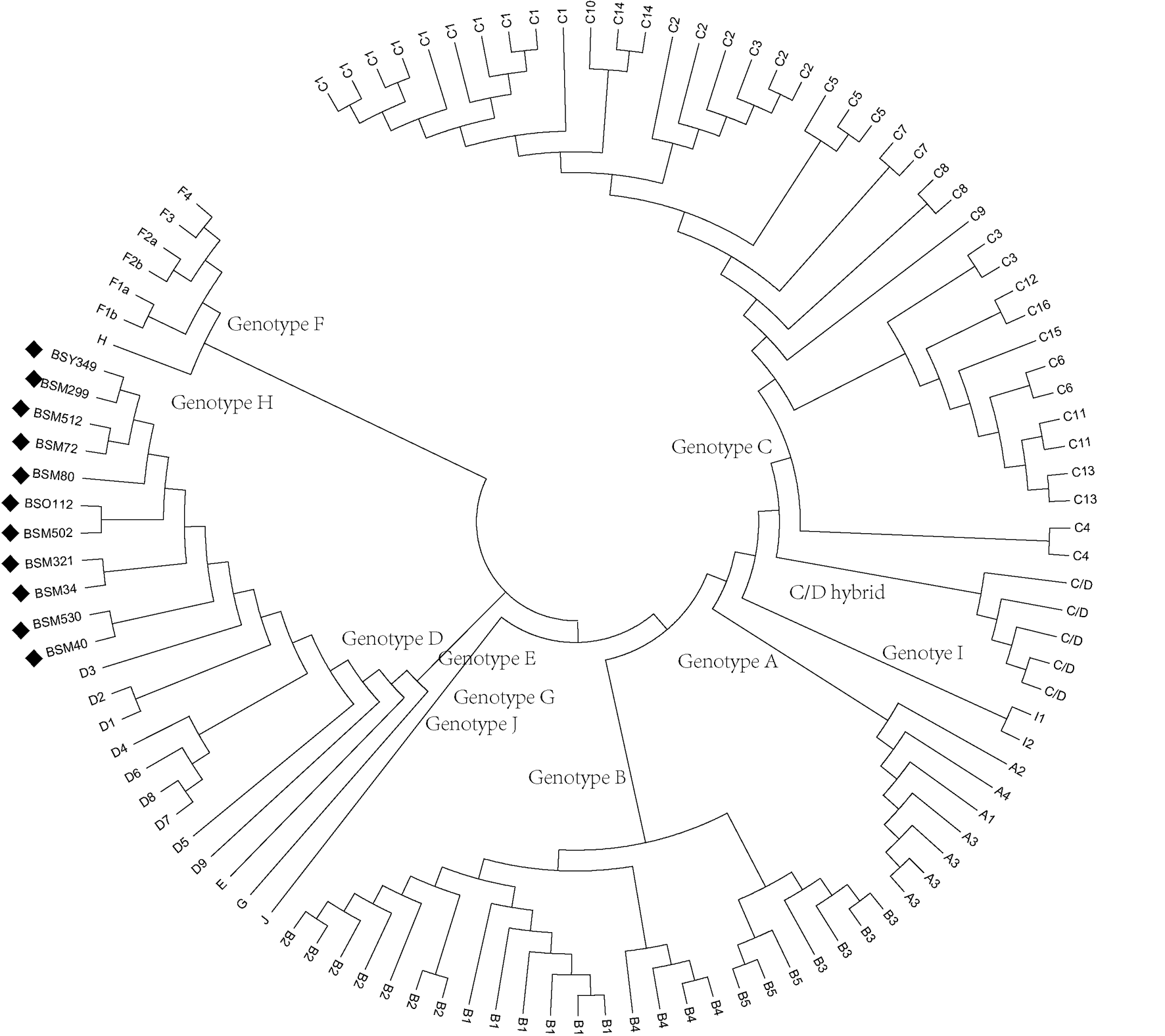

Supplement: Supplementary Figure 1 — Estimated maximum-likelihood phylogeny for Baisha’s Li ethnic HBV whole-genome sequences. The sequences identified in this study are labeled by “◆.” Others are reference sequences. [file Image_1.TIF]
