# Supplementary material for: Molecular Epidemiological Characteristics and Risk Factors for Acquiring HBV Among Li Ethnic in Baisha County, Hainan Island-Subgenotype D3 Was First Discovered in China
Source: Front Microbiol. 2022 Feb 7;13:837746. doi: 10.3389/fmicb.2022.837746 (PMC8859303; doi:10.3389/fmicb.2022.837746)
Supplement: Supplementary file 2 [file Table_1.doc]

Supplementary table 1 Primers used for amplifying partial P region and part of Pre-S/S region

| **Gene** | **Set** | **Primer** | **Sequence (5’-3’)** | **Nucleotide position(nt)** |
| --- | --- | --- | --- | --- |
| P region | 1st | HBV Z | AGCCCTCAGGCTCAGGGCATA | 3046-3066 |
|  |  | HBV 3 | CGTTGCCKDGCAACSGGGTAAAGG | 1163-1140 |
|  | 2nd | HBV P | TCATCCTCAGGCCATGCAGT | 3159-3178 |
|  |  | HBV M | GACACACTTTCCAATCAATNG | 1083-1095 |
| Part of Pre-S/S region | 1st | PF1 | TTATGCCTGCTAGGTTYTATCC | 2635-2656 |
|  |  | S4R2 | AGAAGATGAGGCATAGCAGC | 415-434 |
|  | 2nd | SF3 | TCACCATATTCTTGGGAACAAGA | 2817-2839 |
|  |  | PR4 | CATAGCAGCAGGATGAAGAGGA | 402-423 |

Supplementary table 2 Primers used for identification of HBV whole genomes

| **Set** | **Primer** | **Sequence (5’-3’)** | **Nucleotide position(nt)** |
| --- | --- | --- | --- |
| 1st | WA1 | AGTCAGGAAGACAGCCTACTCC | 3146-3167 |
|  | WA2 | AAAAAGTTGCATGGTGCTGG | 1806-1825 |
| 2nd | WA3 | ATCCGCAGGCCATGCAGTGG | 3194-3213 |
|  | WA4 | CCAATTTATGCCTACAGCCTC | 1777–1797 |
| 1st | WB1 | CTTGAGGCATACTTCAAAGAC | 1695–1715 |
|  | WB2 | TGAGGCATAGCAGCAGGATG | 409–428 |
| 2nd | WB3 | GAGGCTGTAGGCATAAATTGG | 1777-1797 |
|  | WB4 | TGAGAGAAGTCCACCACGAG | 255-274 |
